# From Graphite to Laccase Biofunctionalized Few-Layer Graphene: A “One Pot” Approach Using a Chimeric Enzyme

**DOI:** 10.3390/ijms21113741

**Published:** 2020-05-26

**Authors:** Ilaria Sorrentino, Ilaria Stanzione, Yannig Nedellec, Alessandra Piscitelli, Paola Giardina, Alan Le Goff

**Affiliations:** 1 Department of Molecular Chemistry, University Grenoble Alpes, CNRS, 38000 Grenoble, France; ilaria.sorrentino@univ-grenoble-alpes.fr (I.S.); yannig.nedellec@univ-grenoble-alpes.fr (Y.N.); 2 Department of Chemical Sciences, University Federico II, 80126 Naples, Italy; ilaria.stanzione@unina.it (I.S.); giardina@unina.it (P.G.)

**Keywords:** laccase, hydrophobin, graphene

## Abstract

A chimeric enzyme based on the genetic fusion of a laccase with a hydrophobin domain was employed to functionalize few-layer graphene, previously exfoliated from graphite in the presence of the hydrophobin. The as-produced, biofunctionalized few-layer graphene was characterized by electrochemistry and Raman spectroscopy, and finally employed in the biosensing of phenols such as catechol and dopamine. This strategy paves the way for the functionalization of nanomaterials by hydrophobin domains of chimeric enzymes and their use in a variety of electrochemical applications.

## 1. Introduction

Graphene is a two-dimensional sheet of sp^2^-hybridized carbon that exhibits unparalleled properties such as high planar surface, superlative mechanical strength, and remarkable thermal and electrical conductivity. Due to its extraordinary structure and fascinating properties, graphene is the most studied nanomaterial and can be integrated as the core of cutting-edge devices in many types of applications, ranging from microelectronics to electrochemical energy harvesting systems [1,2,3,4,5,6,7,8]. In biosensing in particular, graphene acts as a conductive platform for biomolecule immobilization and electrochemical detection of bioanalytes [9,10,11,12,13,14]. Graphene-based electrochemical biosensing has relied on the recent developments in the study of graphene electrochemical properties, its production, and biofunctionalization. Different techniques have been investigated for the production of graphene such as scotch-tape transfer, chemical vapor deposition (CVD) growth, and chemically or electrochemically reduced graphene oxide. These strategies lead to different nanomaterials in terms of size, edge and basal defects, number of layers, and oxygenated defect content. Although CVD produces a large surface of monolayer graphene, soft exfoliation of graphite has also been able to provide low-cost access to few-layer graphene (FLG) dispersions. The dispersion stability is one of the main challenges to address during the exfoliation procedure, as in general, the re/aggregation of exfoliated material is minimized by using organic solvents or surfactant–water solutions [15,16,17]. Like other nanomaterials, graphene is a very suitable platform for enzyme immobilization thanks to its high surface area, dispersion in solution, and tunable surface chemistry. Intense efforts have been devoted to this research field in the last five years, resulting in the immobilization of different enzymes for various applications [18]. However, the hydrophobic interactions driving the direct immobilization of active proteins on graphene surface are often difficult to achieve, and modifications of the protein 3D structures can occur with detrimental effects on their functionality [19]. Two main approaches have been implemented, and often combined, to overcome this issue, such as the use of graphene oxide (GO) whose surface is more hydrophilic, or the exploitation of graphene-based composites, such as microcellulose, chitosan, and various metal oxides [18]. 

Recently, proteins prone to forming amyloid structures have been proven to be able to biofunctionalize graphene. In this respect, the fungal self-assembling class I hydrophobin Vmh2 from *Pleurotus ostreatus* has been successfully exploited to disperse and stabilize FLG in ethanol–water mixtures by ultrasonic wave exfoliation. [20] Hydrophobins (HFB) are a family of small self-assembling proteins produced by filamentous fungi, and can be divided into two classes that differ for the nature of the amphipathic layers that they form. Fibrillar structures formed by class I HFB are extremely robust, are disassembled only in strong acids, and share structural properties with amyloid fibrils [21]. HFB efficiently adheres to several hydrophobic surfaces, including 2D materials, such as graphene [20]. This ability has been further exploited by genetic fusion of the hydrophobin to biotechnologically relevant proteins that can be immobilized on various surfaces, obtaining the so-called “self-immobilizing” proteins/enzymes [22]. Recently a new chimeric protein was designed to combine the HFB Vmh2 with a laccase enzyme (Lac), Lac-Vmh2 [23]. Laccases (*p*-diphenol-dioxygenoxidoreductases; EC 1.10.3.2) are multicopper oxidases able to catalyze the oxidation of a wide range of aromatic substrates using oxygen as co-substrate and producing water as the only by-product. These enzymes are promising biocatalysts with possible applications in bioremediation, chemical synthesis, biobleaching of paper pulp, and biosensing [24]. Laccases have been immobilized on various carriers, using different methods with both advantages and drawbacks. Among the various enzymes, the laccase POXA1b from *P. ostreatus* was chosen for its peculiar characteristics such as its stability and activity in a wide range of pHs and temperatures, as well as its high redox potential [25]. The produced Lac-Vmh2 allowed achievement of simple and stable immobilization of the enzyme on polystyrene [23]. 

The main purpose of this work was to advance the biofunctionalization of graphene with Vmh2, immobilizing laccase on FLG by using the fusion protein Lac-Vmh2 through a “one-pot” approach. The presented method is easy, eco-friendly, and versatile, because, in principle, a wide variety of different HFB chimera proteins can be used in this one-pot exfoliation/functionalization procedure. As a proof of concept, the as-prepared Lac-Vmh2/FLG was used for the modification of Glassy Carbon (GC) electrodes to build an electrochemical sensor for phenolic compounds, such as catechol, a well-known environmental pollutant, and dopamine, a renowned neurotransmitter.

## 2. Results and Discussion

### 2.1. Laccase Immobilization on FLG

Graphite exfoliation was carried out, as previously described, by exposing mixtures of Vmh2 protein and graphite to ultrasonic waves [20]. Vmh2-exfoliated graphene is generally stable in 60% ethanol (EtOH) thanks to the presence of the HFB. On the other hand, enzymes are usually used and stable in aqueous buffers. Thus, conditions have to be assessed to preserve both the stability of graphene dispersion and the enzyme activity. The wild-type PoxA1b laccase and Lac-Vmh2 were dialyzed toward different ethanol concentration (20%, 40%, 50% and 60% EtOH,) and 10 mM Tris-HCl pH 8, in order to test the stability of the enzyme in these conditions. Concomitantly, the Vmh2 graphene samples in 60% EtOH were centrifuged and pellets were solubilized with or without the addition of 0.05 mg mL^−1^ of Vmh2 in the same conditions tested for enzymatic stability. The optimal condition for the graphene stability was 50% EtOH + Vmh2 and 40% EtOH + Vmh2 (Figure S1). On the other hand, the enzyme stability was reasonable up to 40% EtOH (Table S1). Thus, looking both at the enzyme and the graphene stability, the 40% EtOH was selected as the optimal solvent for graphite exfoliation in the presence of laccase.

**Figure 1:**
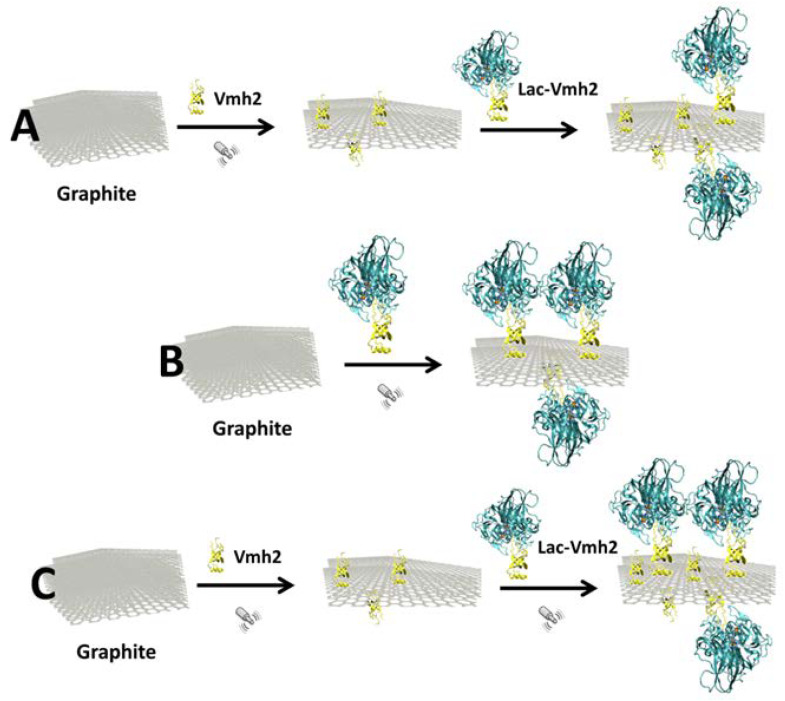
Tests performed to exfoliate and biofunctionalize graphite with laccase chimera, Lac-Vmh2. (**A**) graphite exfoliation in the presence of Vmh2 (~5 h) and subsequent addition of Lac-Vmh2; (**B**) graphite exfoliation in the presence of both Vmh2 and Lac-Vmh2 (~5 h); (**C**) graphite exfoliation in the presence of Vmh2 (~5 h) and addition of Lac-Vmh2 in the last 10 min of exfoliation.

To study and optimize the condition of laccase immobilization on the FLG, several tests were performed (Figure 1). Addition of the enzymes (wild-type or chimera) was performed after exfoliation of graphite in the presence of Vmh2 (route A). Addition of the enzyme solutions to graphite powder was performed at the beginning of the exfoliation (route B). Or, in situ exfoliation of graphite with Vmh2 was followed by the addition of the enzymes in the last 10 min of sonication (route C). Each route was compared by measuring the enzymatic activity [25] of the biofunctionalized FLG (previously separated after centrifugation at 13,000 rpm for 15 min) obtained after centrifugation. Results are displayed in Table S2. Route A led to final no enzymatic activity, indicating that negligible amounts of enzymes can be immobilized on FLG after exfoliation of graphite with Vmh2. When the chimera was used to exfoliate graphene from graphite (route B), the extensive ultrasonication time led to a complete loss of the enzymatic activity of laccase. To avoid a long exposure of the enzymes to ultrasonic waves, route C was used in the course of this work. In the latter route, wild-type or chimera were added during the last 10 min of the exfoliation process in order to prevent enzyme inactivation. An immobilization yield of 5% and 11%, for PoxA1b or Lac-Vmh2, respectively, was estimated, considering the enzymatic activity before and after immobilization. According to the activity of the immobilized Lac-Vmh2, this corresponded to an enzyme loading of about 0.4 U per mg of FLG (Table 1). Lac-Vmh2 showed a slight increased amount of attached enzyme as compared to the wild-type enzyme. Indeed, POXA1b was able to stick to graphene, as already observed using polystyrene [23]. Nevertheless, the stability of the biofunctionalized graphene obtained with Lac-Vmh2 was higher than that of the wild-type enzyme, in terms of both activity and adhesion (Table 1). 

**Figure 2:**
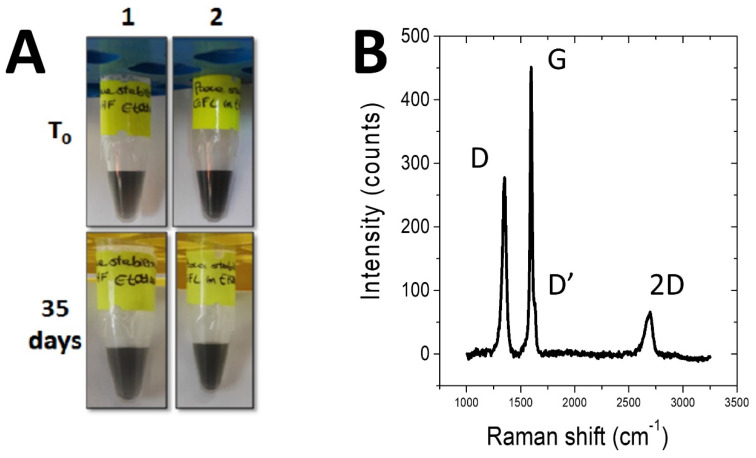
(**A**) Stability of graphene dispersion at time zero and after 35 days: lane1: Graphene/PoxA1b; lane 2: Graphene/Lac-Vmh2. (**B**) Representative Raman spectrum of a Lac-Vmh2-biofunctionlized few-layer graphene (FLG) film.

Raman spectroscopy was also performed in order to characterize the number of graphene layers for these biofunctionalized FLG (Figure 2B). According to Ferrari’s work [26], the high-energy band observed at 2700 cm^−1^ stems for the presence of only few graphene layers (<5 layers), as already observed in the case of the native Vmh2 hydrophobin [20]. It is noteworthy that the Raman spectrum was also performed after several months on the same stock solution of Lac-Vmh2-functionalized FLG without showing any restacking nor aggregation phenomena.

### 2.2. Exploitation of Biofunctionalized FLG in Electrochemical Biosensing

GC electrodes were then modified by drop-casting a solution of biofunctionalized FLG. The incubation was performed at room temperature until the electrode was completely dry, and then several washes with citrate-phosphate buffer pH 5.0 were executed to eliminate the unbound sample.

**Figure 3:**
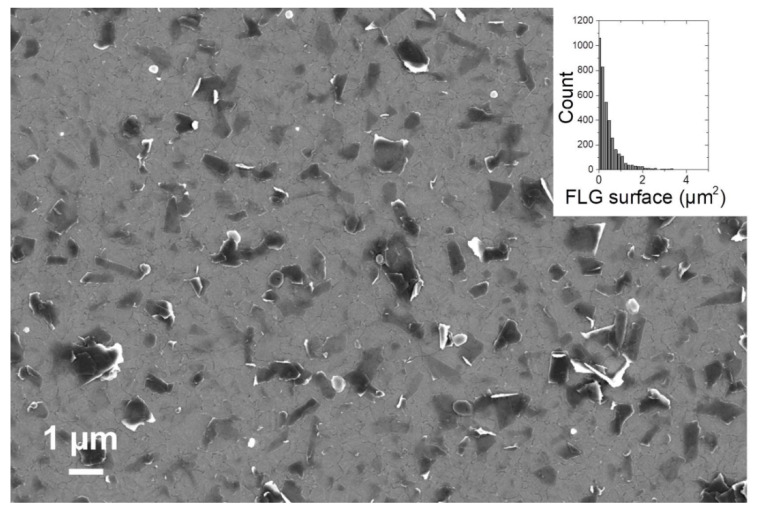
SEM image of biofunctionalized FLG at the surface of a gold electrode; (inset) size distribution of biofunctionalized FLG obtained by Image.

Figure 3 displays a typical SEM image of biofunctionalized FLG deposited onto planar gold electrode. This underlines the homogenous dispersion of FLG both in solution and at the surface of the electrode. According to size distribution study, the sizes of biofunctionalized FLG are mostly below 2 µm^2^. These nanostructured bioelectrodes were employed for biosensing experiments. The principle of laccase biosensors is based on the enzymatic oxidation of phenols or o-diphenols into quinones, the latter being subsequently reduced at the nanostructured electrode polarized at a redox potential required for the electroreduction of quinone into phenols, that is, E = −0.2 V vs. saturated calomel electrode (SCE). The regeneration of the catechol derivative triggers an amplification cycle of “enzymatic oxidation/electrochemical reduction”, which increases biosensing sensitivity.

**Figure 4:**
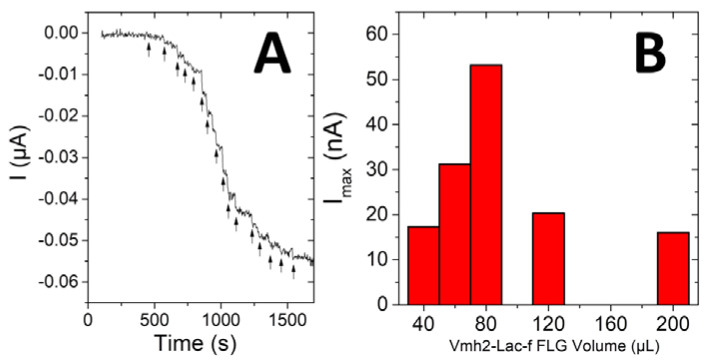
(**A**) Chronoamperometry performed at Lac-Vmh2-biofunctionlized FLG electrode after successive additions of catechol (indicated by the arrows, applied potential = −0.2 V vs. saturated calomel electrode (SCE), 0.1 M Phosphate-buffered saline (PBS), pH 6, 25 °C). (**B**) Plot of the maximum current towards volume of drop-coated Lac-Vmh2-biofunctionlized FLG solutions.

Addition of both catechol and dopamine was monitored at Lac-Vmh2-biofunctionalized FLG electrodes. Figure 4A displays a representative chronoamperometry experiment performed upon the addition of catechol.

Chronoamperometry at different volumes of FLG (Figure 4B) were performed, showing that an optimum value of biofunctionalized FLG was reached after the drop-coating of 80 µL (16 mU) solution on GC electrodes. These experiments underlined the fact that an optimal volume of FLG was needed in order to maximize the amount of immobilized enzymes while also providing efficient diffusion of catechol into the nanostructured FLG conductive film. In order to indirectly confirm the interaction between the Vmh2 domain of the chimera and graphene layer, the wild-type POXA1b was also used in the exfoliation process of FLG. However, on the contrary to the Lac-Vmh2-biofunctionlized FLG electrode (Figure 4A), negligible current was observed for catechol oxidation, underlining the fact that less adsorption of POXA1b was observed on FLG. This also demonstrated the important role of the Vmh2 domain in order to immobilize laccase at the surface of FLG.

**Figure 5:**
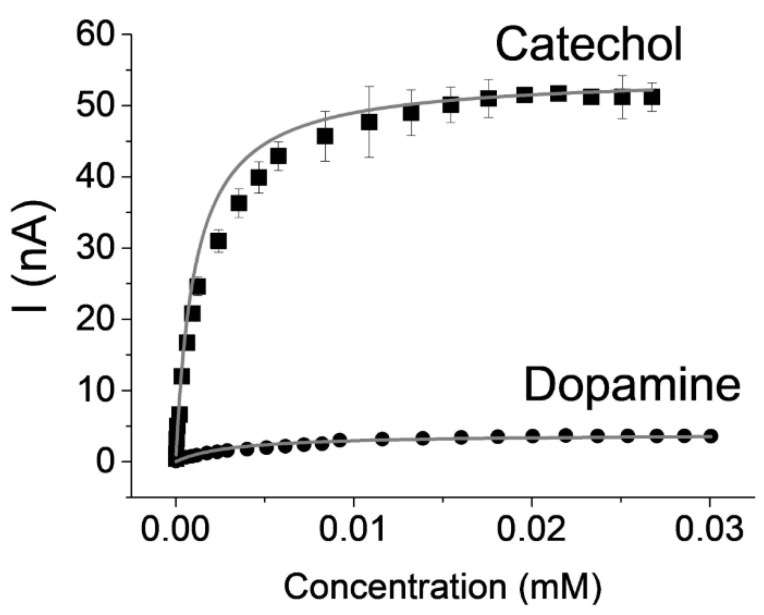
Plot of the catalytic current towards increasing concentrations of (■) catechol and (●) dopamine for electrodes (measurements performed by chronoamperometry at E = −0.2 V vs. SCE, 0.1 M PBS, pH 6, 25 °C).

Figure 5 displays the calibration curves for catechol and dopamine recorded at these electrodes. The shape of the curves was governed by the enzymatic reaction that was reliability modelized according to typical Michaelis–Menten kinetics. Table 2 summarizes the electrochemical characteristics of the modelized curves.

The apparent Michaelis–Menten constant (K_Mapp_) reflected the enzyme-substrate affinity, which was higher in the case of dopamine when compared to catechol for laccase. A lower Km value is advantageous in the case of laccase-based sensors in order to observe linear range with a minimal limit of detection and a maximal sensitivity. Laccase has a better affinity for catechol compared to dopamine. This is the reason why biosensors based on laccases always exhibit better performance towards catechol as compared to dopamine.

**Figure 6:**
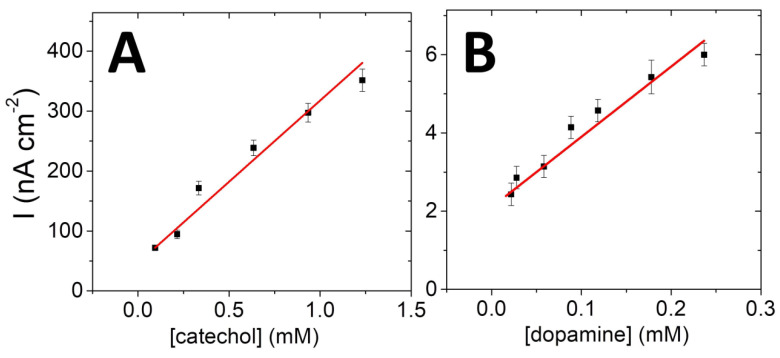
Plot of the linear part of the catalytic current density towards concentration of (**A**) catechol and (**B**) dopamine (E_applied_ = −0.2 V vs. SCE, 0.1 M PBS, pH 6, 25 °C).

The linear part of the curve is shown in Figure 6.

Limit of detection (LOD) of 20 µM was separately measured for both catechol and dopamine, with sensitivity of 0.27 mA M^−1^ cm^−2^ (*R^2^* = 0.97) towards catechol and 16.4 µA M^−1^ cm^−2^ (*R^2^* = 0.96) towards dopamine being measured with respective linear ranges of 20 to 1000 µM and 20 to 250 µM. Although the most efficient phenolic biosensors are based on a combination of tyrosinases and laccases, [27,28] or based on the use of redox hydrogels [29,30], this type of nanostructured biosensor approaches the performances of other types of biosensors that associate graphene and laccases [29,31,32].

## 3. Materials and Methods

All products were purchased from Sigma-Aldrich and were used without further purification. All solvents were of analytical grade. Distilled water was passed through a Milli-Q water purification system to obtain ultrapure water at 18.2 MΩ cm^−1^. Phosphate-buffered saline (PBS) solution was prepared from Milli-Q water.

### 3.1. Electrochemical Measurements

The electrochemical experiments were carried out in a three-electrode electrochemical cell using Ametek Multipotentiostat Princeton Applied Research (Wokingham, UK). A Pt wire was used as the counter electrode and the saturated calomel electrode (SCE) served as the reference electrode. All experiments were conducted at room temperature. All simulated curves were obtained via Origin Pro 9.0. Error bars were estimated from three measurements recorded per sample.

### 3.2. Laccase Enzymes

Both fusion proteins Lac-Vmh2 and wild-type enzyme POXA1b were produced and secreted by the yeast *Pichia pastoris* in the culture media. The supernatant, after centrifugation for 15 min at 6000 rpm at 4 °C, was concentrated and dialyzed towards 50 mM Tris-HCl buffer, pH 8.0, using Centricon Centrifugal Filter Units 10kDa (Merck, Darmstadt, Germany). The laccase enzymes are used without additional purification steps. The total protein concentration was determined using the Pierce 660 method (Thermo Fischer Scientific, Waltham, Massachusetts, MA, USA) and using (Bovine Serum Albumin) BSA as the standard. The laccase activity was assayed at room temperature, monitoring the oxidation of ABTS (2,2′-azino-bis(3-ethylbenzothiazoline-6-sulphonic acid)) at 420 nm (ε_420 nm_ = 3.6 × 10 ^4^ M^−1^ cm^−1^)—the assay mixture contained 2 mM ABTS and 0.1 M sodium citrate buffer, pH 3.0.

### 3.3. In Situ Exfoliation of Graphite

Graphite powder, 1 mg/mL (Aldrich, 332461, mesh number of grains +100, >75%), was exfoliated in batches of 7.5 mL of 60% *v/v* ethanol (EtOH) in MilliQ water (in 20 mL flasks) and 7.5 mL of 50 μg/mL Vmh2, using a medium power tip sonicator (Ultrasound SONOPLUS HD3200, maximum power 200 W, working frequency 20 kHz, KE-76 probe, running at 15% amplitude. BANDELIN, Berlin Germany) and cooling the system in an ice bath. The exfoliation was stopped when the energy value was 450 KJ (about 5 h of sonication time). To remove the unexfoliated material, consecutive 40 min centrifugations at increased centrifugal force were performed (40×, 160× and 620× *g*). We characterized and used the dispersions obtained after the last centrifugation.

Three types of experiments were tested with the laccase enzymes:

- A 7 mL solution of PoxA1b or Lac-Vmh2 in 40% EtOH was added to Vmh2-exfoliated graphene and incubated at 4 °C whilst stirring continuously.
- The immobilization was performed by adding the wild-type or chimeric enzyme solution to graphite powder at the beginning of the exfoliation.
- The wild-type or chimeric enzyme solution was added during the last 10 min of exfoliation. Indeed, the inactivation of the enzyme when higher sonication time was used has been previously verified. The process was performed normalizing the activity units (4 U_tot_ for both) between wild-type POXA1b and Lac-Vmh2 (0.16 mg and 0.44 mg, respectively).

## 4. Conclusions

This work shows that Lac-Vmh2 chimera enzyme can be used both as a surfactant of FLG while also providing enzymatic activity to biofunctionalized nanomaterials. This original biofunctionalization technique represents a soft and biocompatible technique compared to the use of more classic long-alkyl-chain surfactants, leading to stable exfoliation of FLGs. The possibility to merge Vmh2 with active enzymes into chimera enzymes brings novel physico-chemical properties to this exfoliation technique. Here, deposition of these biofunctionalized FLGs on electrodes affords the fabrication of catechol and dopamine biosensors. This novel strategy of functionalizing carbon nanomaterials with specific chimeric enzymes paves the way for the development of many types of novel chimeric enzymes that can be developed for a variety of applications involving multienzymatic systems and biofunctionalization of nanomaterials.

## Figures and Tables

**Table 1:** Summary of the sample enzymatic activity and their characteristics.

Samples	Units in Milligrams of Graphene (U ^mg−1^)	t_1/2_ (Days)	Activity after Washing
Graphene/PoxA1b	0.3 ± 0.1	17	Stable up to the second wash
Graphene/Lac-Vmh2	0.4 ± 0.1	26	Stable up to the fourth wash

The biofunctionalized graphene was stable at least for 30 days (Figure 2A).

**Table 2:** Electrochemical characteristics obtained after modelization of the curve according Michaelis–Menten kinetics; equation: I = (I_MAX_ × [substrate])/(K_M_ + [substrate]).

Substrate	K_M_ (mM)	I_MAX_ (nA cm^−2^)	*R^2^*
Catechol	1.1	775.7	0.99
Dopamine	3.0	55.5	0.95

## Supplementary Materials

Supplementary materials can be found at https://www.mdpi.com/1422-0067/21/11/3741/s1. Figure S1: Stability of graphene dispersion in different solvents; Table S1: t_1/2_ of laccases in different buffers; Table S2: Summary of the immobilization experiments.
